# Editorial: Nonalcoholic fatty liver disease therapy: Exploring molecular mechanisms of well-defined composition from natural plants

**DOI:** 10.3389/fphar.2022.1006750

**Published:** 2022-09-20

**Authors:** Wenji Zhang, Menghao Huang, Runping Liu

**Affiliations:** ^1^ Guangdong Provincial Engineering & Technology, Research Center for Tobacco Breeding & Comprehensive Utilization, Key Laboratory of Crop Genetic Improvement of Guangdong Province, Crops Research Institute, Guangdong Academy of Agricultural Sciences, Guangzhou, China; ^2^ Indiana University School of Medicine, Indianapolis, IN, United States; ^3^ Beijing University of Chinese Medicine, Beijing, China

**Keywords:** nonalcoholic fatty liver disease, Natural plants, NAFLD, NASH, Well-defined Compositio

Nonalcoholic fatty liver disease (NAFLD), a global public health problem in recent years with an incidence rate of approximately 25%, may bring a progressive disease of non-alcoholic steatohepatitis (NASH), cirrhosis, or even hepatocellular carcinoma (Fan et al., 2017; Younossi, 2019). Ever greater attention has been paid to treating NAFLD, and except for the crucial intervention of lifestyle modification, the treatments of drugs and health products have become particularly important in our current world of fast-paced modern life (Zhu et al., 2020). At present, the main adjuvant clinical therapies usually include pioglitazone and vitamin E treatment, but the applicability of these is weak because the long-term effects have yet to be determined (Majumdar et al., 2021). So far, no drugs specifically targeting NAFLD have been approved by the FDA. People have been exploring drugs to treat NAFLD over the past two decades.

Substances from plants including tea, flaxseed, cinnamon, silybin, soy, ginger, and licorice are playing an increasingly promising role in the treatment and prevention of NAFLD (Yan et al., 2020). However, previous research was much more focused on plant-derived extracts or mixtures, which due to the unclear constituents or the lack of standardized product progress, is still a long way from drug development. Therefore, this Research Topic, Nonalcoholic Fatty Liver Disease Therapy: Exploring Molecular Mechanisms of Well-defined Composition from Natural Plants, is inclined to and encourages pharmacological research about NAFLD using well-defined compositions. We hope to discover and collect novel natural compounds, active ingredients, combination formulas, or prescriptions in plants with therapeutic selectivity that can be used for NAFLD, or NASH. Meanwhile, the novel discovery of molecular pathogenic mechanisms of NAFLD studies was also reported.

In this Research Topic, the aurantio-obtusin from Cassia semen (Zhou et al.), pterostilbene from blueberries and grapes (Tan et al.), the combination of bicyclol from *Schisandra chinensis* and berberine from *Coptis chinensis* and *Berberis vulgaris* (Li et al.), artemether from artemisinin (Xu et al.), scoparone from Artemisia scoparia Waldst. etKit and Artemisia capillaris Thunb. (Jiang et al.), nootkatone from Alpiniae oxyphyllae Fructus (Yong et al.), Limonin from lemon (Wang et al.) and theaflavin-3,3′-digallate from black tea (Zhou et al.), attenuated NAFLD/NASH mainly by regulating lipid metabolism and liver inflammation to varying degrees. There are also a lot of new pathways involved. For example, Aurantio-obtusin ameliorates hepatic steatosis *via* AMPK/autophagy- and AMPK/TFEB-mediated suppression of lipid accumulation; bicyclol enhanced lipolysis and β-oxidation through restoring the p62-Nrf2-CES2 signaling axis and p62-Nrf2-PPARα signaling axis, respectively; scoparone downregulated the activation of JNK/Sab signaling, improved hepatosteatosis and inflammation, especially mitochondrial dysfunction; theaflavin-3,3′-digallate was speculated to attenuate leptin-deficient induced NAFLD via Fads1/PPARδ/Fabp4 axis. In particular, berberine and theaflavin-3,3′-digallate also protected NAFLD *in vivo* through regulating gut microbiota which is a hot issue of concern, for example, berberine enriches lipid metabolism-related Bacteroidaceae (family) and *Bacteroides* (genus); theaflavin-3,3′-digallate increased the abundance of Prevotellaceae_UCG-001, norank_f_Ruminococcaceae, and GCA-900066575 and significantly decreased that of Parvibacter. Noticeably, with the multiple targets, the ganweikang tablet (Ma et al.), based on traditional Chinese medicine theory and clinical experience, was verified to improve NAFL and NASH by modulating inflammation, apoptosis, and fatty acid oxidation by inhibiting NFκB, caspase-8, and activating PPARα. In addition, artemether, the combination of bicyclol and berberine, and pterostilbene, also mediated the development of liver fibrosis *in vivo*, which protects liver injury more broadly. These results have a direct impact on the treatment of NAFLD and provided promising candidates for its therapy.

The pathophysiological mechanisms of NAFLD is a complex, “multiple-hit theory” that has gradually been posited to explain the pathogenesis of NAFLD, including visceral obesity and lipodystrophy-like phenotype, diabetes, insulin resistance, *de novo* lipogenesis, gut dysbiosis, genetic factors, epigenetic modifications, etc (Zhang et al., 2022). Great progress has been made on the alleviating effect of plant-derived composition on fatty liver disease, but the research on its mechanism is not comprehensive and in-depth, and its clinical application needs to be evaluated based on the “multiple-hit theory”.
